# A two-armed pragmatic randomized controlled trial comparing the effectiveness of two self-compassion interventions at reducing perceived stress

**DOI:** 10.3389/fdgth.2026.1680033

**Published:** 2026-02-09

**Authors:** Lina S. Kalon, Leif Boß, Carmen Wiencke, Anna-Carlotta Zarski, Dirk Lehr

**Affiliations:** 1 Department of Health Psychology and Applied Biological Psychology, Institute for Sustainability Psychology, School of Sustainability, Leuphana University of Luneburg, Luneburg, Germany; 2 Institute for Interactive Systems, Technische Hochschule Lubeck, Lubeck, Germany; 3 Psychological Counselling Service, Department of Studies and Teaching, University of Hamburg, Hamburg, Germany; 4 Department of Clinical Psychology, Division of eHealth in Clinical Psychology, Philipps-Universität Marburg, Marburg, Germany

**Keywords:** depression, digital intervention, positive psychology, public mental health, self-compassion, self-criticism, stress

## Abstract

**Introduction:**

A lack of self-compassion has been found to be associated with stress and a variety of psychological disorders. Interventions aimed at fostering self-compassion have been proposed as a promising approach to promoting public mental health. In the context of universal prevention, low-threshold interventions are needed. Against the background of substantial heterogeneity in effectiveness of self-compassion interventions at enhancing mental-health outcomes, the newly-developed guided digital intervention “Namah” and a pre-existing, broadly-available printed workbook as a bibliotherapeutic approach were investigated.

**Methods:**

In a randomized-controlled-trial (*N* = 200), we compared Namah and the above-mentioned workbook, both aiming to reduce stress by strengthening self-compassion in a universal prevention setting. Within- and between-group differences in perceived stress, the primary outcome, and further secondary outcomes were investigated eight weeks and six months after randomization via intention-to-treat analysis (ITT). Reporting follows CONSORT-guidelines. The study was registered in the German Clinical Trial Register (https://drks.de/search/en/trial/DRKS00027552), the approved primary registry of the (WHO) for Germany.

**Results:**

Exploratory analysis for both self-compassion interventions revealed significant and meaningful reductions in stress (*d* = 0.68–0.79), symptoms of depression (*d* = 0.30–0.48) and self-criticism (*d* = 0.40–0.55), as well as increased self-compassion (*d* = 0.54–0.63) within each group. However, during between-group analyses, ITT ANCOVA revealed no significant differences either at eight weeks post-intervention (*d* = 0.13) or at 6-month follow up (*d* = 0.14) for perceived stress or any secondary outcome. Various sensitivity analyses corroborated these findings.

**Discussion:**

Two distinct low-threshold approaches to fostering self-compassion seem beneficial for reducing stress and symptoms of depression. Although superiority of the guided digital intervention was expected, results suggested that both the digital and printed bibliotherapeutic formats are valuable candidates for mental health promotion in the general population, given the interventions are clearly structured, behaviorally oriented and provide at least a minimal regular human contact.

**Clinical Trial Registration:**

https://drks.de/search/en/trial/DRKS00027552, identifier DRKS00027552.

## 1 Introduction

The World Health Organization (1) stated that chronic stress has emerged as one of the most significant health threats of the 21st century. Data from the World Health Survey found a linear increase in the 12-month prevalence of depressive disorder with higher perceived stress scores (2), an average prevalence of 6.2% of depression in low- and middle-income countries and a similar 12-month prevalence of major depressive disorder of 8.2% in a high-income country like Germany (3). The life-time prevalence varies considerably between countries and was found to be highest in European countries, at an average of 11.3%. In terms of burden, depressive disorders accounted for the highest proportion (37.3%) of disability-adjusted life years due to mental disorders (4) and were ranked 11th among all causes of disability adjusted life-years in 2023 (5). Regarding potentially modifiable risk factors that provide targets for prevention, it is important to note that reducing stress in just one life domain, particularly in the context of perceived stress resulting from the work-life domain, could alone prevent 17.9% of depressive disorders globally (6).

Prolonged stress also predisposes one to physical health problems that include increased inflammation and cardiovascular heart disease (7). At the same time, there has been an increase in stress-related sickness and absenteeism in recent years (8, 9). Taken together, chronic stress and stress-related pathology are of concern not only with regard to individual suffering but also for society. Although several approaches exist to tackling stress [e.g., (10)], broadening the repertoire of effective interventions seems prudent to address the high societal burden of stress, its various causes, and individual preferences in the interventions used—particularly in an increasingly diverse population. One promising route seems to address self-compassion, the manner of relating to ourselves in general and especially in times of stress or suffering, as one of the most impactful resilience factors (11), to avoid spiraling downward towards chronic stress. This also ties in with Cuijpers' (12) call for indirect, less stigmatizing or non-pathologizing interventions to improve public mental health. Approaches that also focus on engaging with positive aspects and building personal resources as resilience factors may be perceived as more appealing than those aimed primarily at reducing deficits like low levels of problem-solving skills, limited coping capacity, or difficulty in managing challenges in general.

With the rise of new developments to promote mental health, including positive psychological approaches, self-compassion has been emphasized as a personal resource that helps to combat maladaptive coping with stress (13). Conversely, high tendencies toward self-criticism have been linked to psychological distress (14) and a variety of mental disorders including depression (15), social anxiety (16), borderline disorder (17), posttraumatic stress disorder (18) and eating disorders (19). As such, high levels of self-criticism may play a role as a potential transdiagnostic factor.

An overly self-critical attitude can be described as a process of negative self-evaluation causing stress and preventing adaptive coping strategies (13, 20, 21). Self-compassion is believed to act as a buffer against the detrimental effects of elevated levels of self-criticism (22, 23). In their meta-analysis of 20 randomized controlled trials (RCTs), Wakelin and colleagues (21) found support for the efficacy of self-compassion interventions at reducing self-criticism in both clinical and nonclinical samples. There is increasing interest in self-compassion as a strategy to address self-criticism and the stress associated with it (21). In this context, self-compassion refers to the way we relate to ourselves in times of suffering, failure, or inadequacy (24, 25).

Neff (24, 26) conceptualized self-compassion as a multifaceted construct that encompasses three dimensions and six opposing components, each representing a specific way in which individuals relate to themselves.

The first dimension of self-compassion refers to the opposing components of self-kindness and self-judgment. It concerns the emotional stance people take toward themselves, especially in times of stress or suffering. Individuals high in self-compassion respond with warmth, understanding, and self-kindness, whereas those low in self-compassion tend to react with self-judgment, harsh criticism, or feelings of frustration about their perceived shortcomings.

The second dimension addresses common humanity as opposed to isolation and is about the cognitive interpretation of one's fallibility. The common humanity perspective involves recognizing that imperfection, mistakes, and setbacks are part of the broader common human experience. In contrast, low self-compassion is characterized by isolation, where individuals feel that their struggles are unique, setting them apart from others and reinforcing a sense of separateness.

The third dimension pertains to attentional regulation, e.g., in the face of failure and ranges from mindfulness to overidentification. Here, self-compassion involves adopting a mindful, balanced awareness of one's imperfection—acknowledging weaknesses without suppression or exaggeration. Its opposite is overidentification, where individuals become entangled in their negative emotions, ruminating on them to an extent that amplifies their distress.

Taken together, this illustrates that self-compassion can be understood as a continuum, consisting of three dimensions with opposing poles: self-kindness vs. self-judgment, common humanity vs. isolation, and mindfulness vs. overidentification. According to Neff (24, 26) higher self-compassion reflects a coherent pattern of emotional warmth, connectedness, and mindful awareness, whereas lower self-compassion represents a constellation of self-critical, isolating, and negative biased tendencies. The widespread use of Neff's measure of self-compassion (27) has caused her conceptualization to become highly influential.

Beyond reducing self-criticism, meta-analytic evidence also supports the efficacy of self-compassion interventions in reducing stress (27, 28). Evaluating RCTs that included both digital and analog interventions aimed at strengthening self-compassion, Ferrari et al. (27) reported an effect (*g* = 0.67) on stress across different comparison groups which is in accordance with the findings of Han and Kim (28), both post-intervention (*SMD* = 0.43) and at follow-up (*SMD* = 0.23).

Whereas lacking self-compassion is stated to be a vulnerability factor for depression (29) meta-analytically derived results concerning the efficacy of strengthening self-compassion for reducing depressive symptoms are not homogeneous so far (28, 30). Wilson and colleagues (30) conclude a comparable effectiveness to other interventions. The authors have not found moderating effects of symptom severity including study populations with a range of clinical and subclinical mental health problems meta-analytically. Contrasting this, Mistretta et al. (31) meta-analytically revealed in studies using samples of patients with chronic pain a reduction of depressive symptoms (*SMD* = 0.29) by self-compassion interventions compared to control. This is consistent with results from the meta-analysis of Millard et al. (32), who showed an effect of *d* = 0.24–0.25 on depressive symptoms in a clinical sample by self-compassion interventions compared to both: waiting lists and other interventions.

Digital formats have the potential to make self-compassion interventions especially accessible and, as such, more widely available. They offer further advantages like in terms of scalability, personalization, and flexibility regarding where the training is carried out, at what time, and at what pace the exercises are done, thereby making a valuable contribution to mental health promotion. On subgroup analysis, Han and Kim (28) identified 23 RCTs evaluating digital self-compassion interventions, and found support for their potential to reduce stress and symptoms of depression. To the same direction point meta-analytic findings of Linardon (33) that underscore the potential of mindfulness- and compassion-focused mobile interventions on distress.

However, low-threshold formats are not limited to digital interventions, as bibliotherapy has already been identified as a potentially effective approach in early meta-analyses (34, 35). Also, more recent RCTs have revealed bibliotherapeutic formats to be effective at reducing depressive symptoms (36) and anxiety (37). Moreover, in a randomized waitlist-controlled trial, Sommers-Spijkerman et al. (38) showed initial evidence supporting the beneficial effects of a compassion-focused self-help book with e-mail guidance on well-being. Although both the digital and bibliotherapeutic formats have several characteristics in common—like, working on one's own schedule and having flexible times and locations—bibliotherapy may appeal to those who are fatigued by an overly-digitalized life. In recent years, the range and variety of available self-help books seem to have become increasingly diverse and widespread, indicating broad interest in them among the general public. Recently, utilizing existing and widely-available self-help materials has been advocated as a comparator to mimic real-world conditions in trials (39, 40). Against this background, to the best of our knowledge, no RCT has yet been published that has compared the effectiveness of a bibliotherapeutic self-compassion approach against that of a digital self-compassion intervention as two potential low-threshold formats to reducing stress in the general population.

The overall objective of the current RCT was to investigate the effectiveness of a newly-developed digital self-compassion intervention (Namah) compared to bibliotherapy in the form of an established self-compassion workbook as two formats of low-threshold interventions. The study focuses on the effects on perceived stress, as conceptualized by Cohen et al. (41), who proposed that stress reflects the degree to which individuals find their lives unpredictable, uncontrollable and overloaded. Their conceptualization captures the global experience of stress, independent of its specific causes, and is therefore well suited to the present intervention, in which individuals should learn to apply self-compassion to all life domains. First, the study examines whether the interventions differed in their effectiveness in reducing perceived stress eight weeks after randomization as the primary outcome and six months after randomization. Secondary outcomes included depressive symptoms and different associated risk factors including self-criticism and negative emotions as well as resources like self-compassion, mindfulness, positive emotions, satisfaction with life and well-being both eight weeks and six months after randomization.

## 2 Materials and methods

### 2.1 Design

With the aim to compare two low-threshold interventions based on different formats to strengthen self-compassion, a two-armed RCT was conducted. Online surveys were completed in both groups three times: at baseline (pre-intervention), eight weeks (post-intervention) and six months (6-MFU) after randomization. Randomization took place immediately after the baseline assessment.

The trial was approved by the Ethics Committee at Leuphana University of Luneburg, Germany: EB-Antrag_2021-04_SelbstkritikC4C and registered at DRKS on April 01st, 2022 (https://drks.de/search/de/trial/DRKS00027552), the approved primary registry of the World Health Organization (WHO) for Germany.

Results are reported following CONSORT guidelines (42).

### 2.2 Deviations from protocol

Initially, the inclusion criteria exclusively covered health care workers. However, since the intervention showed relevance beyond the original target group and to accelerate recruitment, its availability was extended to the general population as registered via an amendment on October 21st, 2022 during the recruitment phase. Therefore, all outcomes related to occupational health are summarized in the Supplementary Material 1.

### 2.3 Sample size calculation

Based on previously-published evidence on the effectiveness of similarly-designed digital interventions reducing stress (39, 43) and on transdiagnostic risk factors like repetitive negative thinking (44), we assumed superiority of the digital intervention Namah over the bibliotherapeutic approach. To estimate an adequate sample size, *a priori* power analysis was performed using G*Power (version 3.1). The difference between groups in PSS-10 scores was considered to be practically meaningful if it exceeded 2 points (45). Following considerable deliberation, the research team reached a consensus on 2.5 points as the minimal practically-important difference between groups at post-intervention. Assuming a standard deviation of 6.3 points [representative German standardization sample (46);] resulted in a tested effect of *d* = 0.4. For a two-sided test with 80% power and a significance level of 5%, the targeted sample size was calculated to be *N* = 200. Data from all randomized participants were analyzed, following the intention-to-treat (ITT) principle.

### 2.4 Participants and procedure

Participants were recruited from the general population between April 2022 and May 2024 via social media, acquisition in various companies in Germany, newsletters and flyers. The first participant was enrolled on April 22nd, 2022. Individuals expressing interest in participating were required to register via the study website (https://geton-training.de/) by providing an email address. After that, they received an email with detailed information about the study and eligibility criteria were queried. For this purpose, participants were required to complete a one-page form indicating whether they met various criteria and to return it to the research team via email. With the aim of mimicking universal prevention, inclusion criteria were limited to being 18 years old or older and providing informed consent, with no symptomatic inclusion criteria applied. Exclusion criteria included a diagnosis of psychosis or dissociative symptoms, current psychotherapy or being on a waiting list for such, simultaneous participation in some other health training program designed to reduce stress or promote self-compassion, initiation or change in medication intake due to stress, anxiety or depression within the last four weeks, and the absence of regular internet access. Upon completion of the registration process, individuals who met the eligibility criteria were asked to complete the baseline survey (pre-intervention), using the LimeSurvey scientific online survey tool (https://ps-limesurvey.leuphana.de/). Following completion of the questionnaire, participants were randomly assigned to one of the two study arms using a computer-generated randomization list. A 1:1 randomization ratio was employed, with a block size of 10. To conceal the allocation sequence, the personnel responsible for randomization had no contact with participants and were not involved in conducting the study. While blinding participants to their group allocation was infeasible, they were not informed about the presumed superiority of the digital Namah intervention. Participants were provided immediate access to the intervention to which they had been randomly assigned. All participants allocated to training with the workbook were furthermore provided with access to the Namah intervention after the 6-MFU assessment. Unrestricted access to care-as-usual was possible for both groups throughout the entire study.

### 2.5 Outcome measures

Primary and secondary outcomes were assessed online using validated questionnaires in German. With the aim of capturing the current state of each outcome, we referred to a time frame of the past two weeks in the questionnaire introduction, except for stress and depressive symptoms, for which we adopted the standardized time frame of one week. Data were collected at three time points: baseline (pre-intervention), eight weeks (post-intervention), and again at six months after randomization (6-MFU). The baseline assessment also included collection of socio-demographic variables. At post-intervention, variables pertaining to participants' satisfaction and uptake of co-interventions were assessed.

#### 2.5.1 Primary outcome measure

The primary outcome was the total score on the Perceived Stress Scale [PSS-10; (41)], which consists of 10 items with scores ranging from 0 to 40 and reliability *α* = .84−.86 (41).

#### 2.5.2 Secondary outcomes measures

##### 2.5.2.1 Psychopathology

The Epidemiological Studies Depression Scale [CES-D; (47)] containing 15 items and a score range of 0–45 was used to assess depressive symptoms. A total score of ≥18 suggests clinical levels of depressive symptoms (48). The scale demonstrates excellent reliability with a Cronbach's alpha of .95 (47).

##### 2.5.2.2 Risk-factors and resources

To assess self-criticism, we used two subscales (self-criticizing and self-reassuring) of the Forms of Self-Criticizing/-Attacking and Self-Reassuring Scale [FSCRS; (49)]; *α* = .90) according to recommendations of Halamová et al. (50). 17 items were included (sum range: 0–36 for the self-criticizing subscale, 0–32 for the self-reassuring subscale). In order to reduce the burden on participants, the self-hate subscale was not employed.

To measure positive and negative emotions, the Modified Differential Emotions Scale [mDES; (51)]; *α* = .79−.89), with 20 items and two subscales (positive emotions, negative emotions), was used (sum range: 0–40 per subscale).

The Self-Compassion Scale [SCS; (26, 52); short form (53); with *α* ≥ 0.86 of the short form], which has 12 items and six subscales (self-kindness, common humanity, mindfulness, self-judgement, isolation, over-identification) was used to assess self-compassion (total sum range: 12–60).

The 15 items of the Mindful Attention Awareness Scale [MAAS; (54); *α* = .83] were used to estimate participants' level of mindfulness (sum range: 15–90).

##### 2.5.2.3 Well-being

Not feeling stressed or burdened does not necessarily mean feeling comfortable or experiencing well-being [e.g., (55–57)]. Therefore, as further secondary outcomes, we deemed fruitful not only to examine interventions' effects on deficit-oriented endpoints, but also take the positive end of this dimension into consideration.

The 18-items Psychological Wellbeing Scale Short Version [PWBS; (58); *α* = .86−.93] structured into six subscales (autonomy, mastery, personal growth, positive relations, purpose in life, self-acceptance) was employed to measure different aspects of psychological well-being (sum range: 3–18 per subscale).

Subjective well-being was assessed using the corresponding subscale of the WHO-5 Well-Being Index (59), consisting of five items and a sum range of 5–30 [*α* = .83−.91; (60, 61)].

Life satisfaction was measured utilizing the Satisfaction with Life Scale [SWLS; (62); *α* = .87], with its five items and sum range of 5–35.

##### 2.5.2.4 Client satisfaction

The Client Satisfaction Questionnaire (63)—adapted to digital interventions [CSQ-I; (64); *α* = .93] with eight items and a sum range of 8–32—was used to investigate participants' satisfaction with the training program to which they had been assigned, with scores >23 indicating meaningful satisfaction.

### 2.6 Interventions

Both intervention types are described according to the template for intervention description and replication [TIDieR; (65)].

The Namah group participated in a seven-week digital training program comprising seven sessions of 60–90 min each. Participants were advised to complete one session per week (as an overview see Table 1) and obtained access to the digital training program via a digital platform (https://coach.geton-training.de/), allowing them to take part individually anytime and anywhere they preferred. Additionally, a mobile application provided access to audio files for exercises offered in the digital training program that are suitable for daily practice, especially guided meditations. Instructions for accessing and using the mobile application were provided in the first training module. Trained eCoaches, holding a Master's degree in Psychology and supervised by a clinical psychologist, provided written feedback to participants via the platform after they completed each training module. Every module was unlocked sequentially after participants completed and received feedback for the previous module. To ensure the standardization of feedback, all eCoaches followed a written manual. The number of sessions completed by participants was monitored through the platform's tracking system, providing adherence data. Up to three reminder messages were sent by the eCoaches at five-day intervals via the platform if participants were inactive for more than one week. The participants were provided with free and unlimited access to the intervention components.

**Table 1:** Overview: Namah intervention content.

Session topics	Objectives
Becoming aware of one's own self-criticism	To become acquainted with the training program and the concept of self-compassion, and become aware when criticizing oneself
Developing a friendly approach to oneself	To draw attention to both the functionality and dysfunctionality of self-criticism, and encourage participants to adopt a self-compassionate perspective
Perceiving self mindfully	To learn how a mindful attitude towards oneself helps to strengthen self-compassion
Building a helpful mindset	To learn how to identify self-critical thoughts and convert them into constructive thoughts
Learning to deal with difficult emotions	To cope with difficult emotions and reflect on their appropriateness
Developing self-supportive behavior	To determine what one feels good about
Being a friend to oneself in the future	To transfer what has been learned so far into the future and build on it
Optional modules available throughout the Namah training program	
Personal objectives	To set realistic objectives
Perfectionism	To learn to deal with perfectionist tendencies
Other people's expectations	To learn how to deal with external expectations
Individual strength	To learn to recognize and use one's own strengths
Personal values in life	To discover one's own values and act in accordance with them

The intervention has psychoeducational elements and a variety of exercises. Between the weekly sessions, participants were invited to integrate and practice what they had learned in their everyday lives. As part of the weekly training sessions, participants were instructed in a number of different topics, including the development of a mindful approach to themselves, which involves reducing excessive identification with one's own mistakes and weaknesses. The concept of accepting mistakes as a natural part of the human experience was introduced, as was the importance of discarding perfectionist demands placed upon oneself. Another key topic was the process of relating to oneself in a manner similar to relating to a friend, rather than through the lens of self-judgement. Testimonials from personas were developed to encourage participants and provide examples of how to cope with backlashes or possible obstacles and how to integrate the learned content in everyday life. The training program also included videos, audios, and gamification components and was delivered in an adaptive and interactive manner. Further details are provided in Supplementary Material 4 as a comprehensive explanation of the training components. Supplementary Table 5 in the supplementary materials additionally provides an overview of which exercises were supposed to strengthen each component; self-kindness, mindfulness and common humanity. After its development, the training program was piloted with 11 people and revised based on their feedback. The intervention was neither tailored nor modified during the study period.

After reviewing various bibliotherapeutic formats with the aim of strengthening self-compassion, a book was chosen that is widely available and goes beyond solely psychoeducational elements. As such, it is primarily a workbook that focuses on exercises, since practice was also the central element in the Namah training program. Participants randomized to the workbook training group practiced for seven weeks using the 143-page self-compassion workbook “Der kleine Selbstcoach: Sei gut zu dir selbst” [The Little Self-Coach: Be Kind to Yourself] (66). Aims included discovering and reflecting on different needs, shifting attention from one's deficits to individual strengths, mindfully recognizing oneself, prioritizing self-care activities and translating self-judgment into needs and feelings (see Table 2 as an overview). For more details, please refer to Supplementary Material 6, where the components are described in greater depth. Alongside brief psychoeducational components, a variety of exercises were included and participants were instructed to work through over roughly 60–90 min each week, as well as to apply newly-learned content in everyday life throughout the following week. The comprehensive workbook was vividly and colorful laid out and clearly structured. Once weekly, participants also were sent questions per mail concerning their current level of well-being and how mindfully and compassionately they were treating themselves, equivalent to weekly questions that the Namah group was provided with. At the end of each training program, participants had the option of receiving individualized feedback on their progress based upon their responses to the weekly questions.

**Table 2:** Overview: workbook content.

Section topics	Objectives
Four steps towards self-empathy	To pay attention to oneself and one's own needs
Reflecting on different personality traits and needs	To acquire knowledge regarding the intricacies of one's own identity and acknowledge of the existence of diverse facets within one's personality
Discovering and recognizing one's needs	To acknowledge the importance of one's personal needs
Freeing oneself from unwanted habits	To recognize the need underlying each unwanted habit
Celebrating success and inner strength	To shift attention away from deficits toward strengths
Consolidating the five pillars of self-compassion	To recognize oneself mindfully To take responsibility for one's own needs To prioritize self-care activities To cultivate human dialogue To translate self-judgement into needs and feelings

### 2.7 Statistical analyses

Descriptive statistics for the primary and secondary outcomes are reported, with data collected pre- and post-intervention, as well as at six-month follow-up (6-MFU).

Missing data (29.55% at post-intervention and 43.96% at 6-month follow-up for the primary outcome) were addressed by implementing multiple imputations. 20 complete datasets were generated with plausible values for missing observations, which were then analyzed separately. Results are presented as pooled and adjusted mean values and standard deviations following Rubin's Rule to produce overall estimates and account for the uncertainty due to missing data (67). All statistical analyses were performed in accordance with the intention-to-treat (ITT) approach using R Studio (Version 2022.07.2 Build 576). In all cases, a two-tailed significance level of *p* ≤ .05 was employed.

In accordance with the recommendations of O'Connell et al. (68), between-group differences eight weeks after randomization (post-intervention) and at 6-MFU were derived using analysis of covariance (ANCOVA), with the baseline value of the outcome of interest as a covariate. This is a robust method to account for potential baseline differences, as demonstrated in methodological research by Wang et al. (69), Egbewale et al. (70), and Schminder et al. (71). Furthermore, Cohen's *d* values and corresponding 95%-confidence intervals (*95%-CI*) were calculated for between-group differences in the primary and secondary outcomes post-intervention. Due to baseline differences in depressive symptom severity between the groups, the baseline CES-D score was used as a covariate in the primary ANCOVA.

As both groups received interventions, effects within each group also were analyzed by paired sample t-tests and by calculating within-group Cohen's *d* values using the difference in means at each respective assessment time point and the respective pooled standard deviations.

For sensitivity analyses, various methods were employed to ascertain the robustness of the results obtained via ITT analyses. First, both study completer and intervention completer analyses were conducted with study completers defined as participants who responded to the assessment of perceived stress at post-intervention, while the intervention completer sample only included those who had completed at least six of the seven sessions. Second, linear mixed modeling was performed for the primary outcome as an alternative method to handling missing data (68). Third, to further stabilize the results, questions regarding experiences with other mental health training programs and similar co-interventions were assessed post-intervention and analyzed as covariates. Forth, due to observed baseline differences in depressive symptom severity between groups, the baseline CES-D score was included as a covariate in the primary ANCOVA.

To define response rates, we applied the reliable change index proposed by Jacobson and Truax (72), using equal change scores for both reliable improvement and deterioration of perceived stress and depressive symptoms, resulting in delta values of +/− 4.41 points on the PSS-10 and +/− 4.53 points on the CES-D.

## 3 Results

### 3.1 Participants

**Figure 1:**
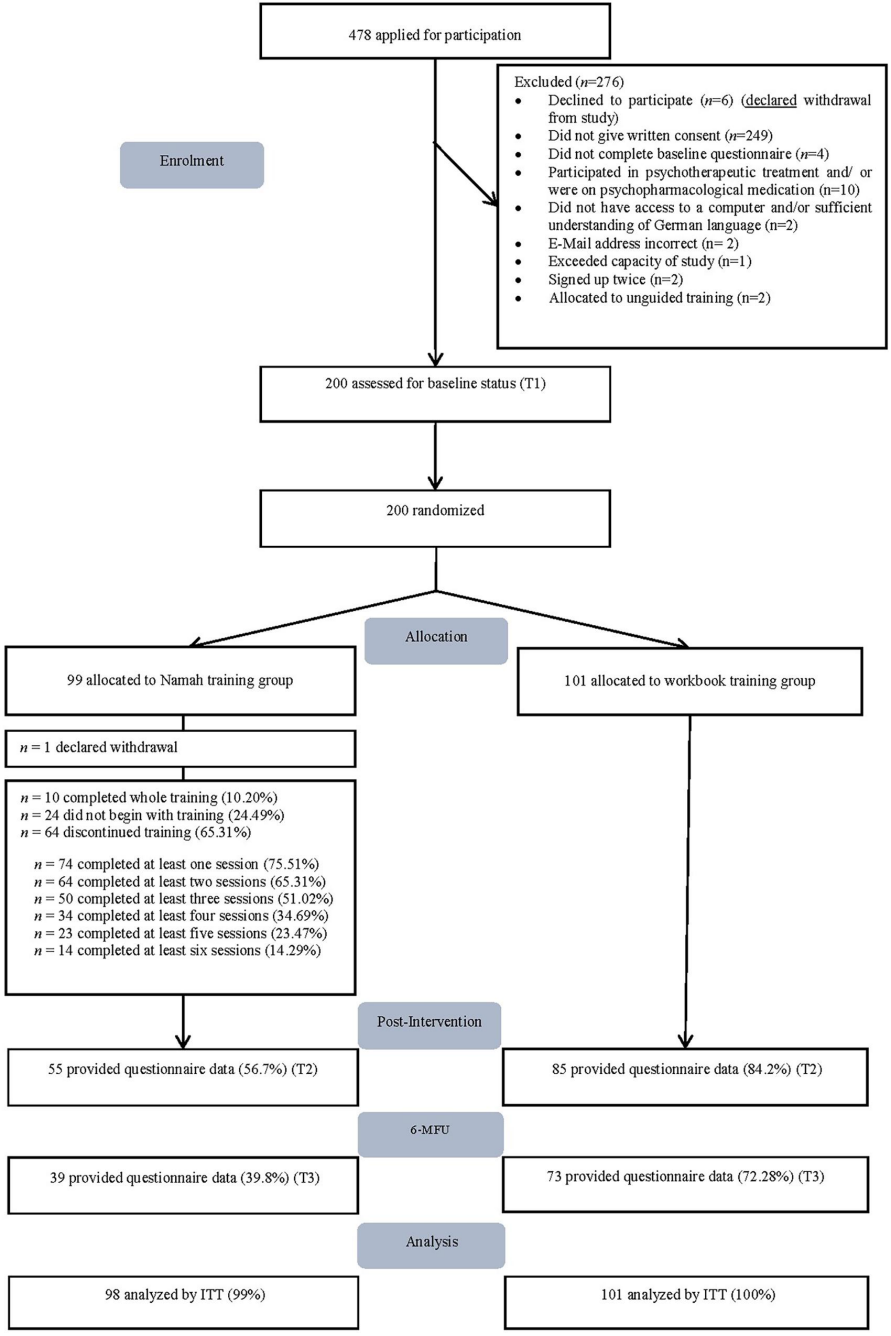
Study flow of the randomized-controlled-trial (RCT). 6-MFU = 6-month-follow-up. ITT, intention-to-treat.

A total of 478 individuals applied for participation, among whom 200 were deemed eligible and completed the baseline assessment. Of these 200, 101 were randomized to the study arm practicing with the workbook whereas 99 were allocated to the study arm training with the Namah program. One participant in the Namah group declared their intention to withdraw and requested data deletion; therefore, ITT analyses were conducted with *N* = 199. Figure 1 illustrates the trial's study flow.

### 3.2 Baseline characteristics

Baseline characteristics are summarized in Table 3. On average, participants were 42 years old (*SD* = 13) and 81.9% were female. Most participants had a university degree (71.4%). Relationship status was almost evenly distributed between being single (42.2%) and married/cohabiting (50.8%) whereas another 7.0% reported to be divorced or separated. Roughly three in four (73.4%) reported some past experience with mindfulness-based interventions. No practically-meaningful between-group differences were observed in demographic characteristics. Overall, 47.0% met the screening criterion for depression, scoring ≥18 points on the CES-D at baseline, though this was not equally balanced between the groups (Namah group: 37.4%; workbook group: 56.8%). Marginal means and standard deviations for all outcomes and assessment points are listed in Table 4.

**Table 3:** Demographic sample characteristics.

Baseline characteristic	Total (*N* = 199)		Namah group (*n* = 98)		Workbook group (*n* = 101)	
	*n*	%	*n*	%	*n*	%
Age (*M/SD*)	41.7	13.3	41.6	13.4	41.8	13.3
Sex						
Men	34	17.1	20	20.4	14	13.9
Women	163	81.9	78	79.6	85	84.2
Other	2	1.0	0	0	2	2.0
Relationship						
Single	84	42.2	44	44.9	40	39.6
Married or cohabiting	101	50.8	45	45.9	56	55.4
Divorced or separated	14	7.0	9	9.2	5	5.0
Education						
No school graduation	1	0.5	1	1.0	0	0
Secondary school certificate	13	6.5	5	5.1	8	7.9
A-level	43	21.6	14	14.3	29	28.7
University degree	142	71.4	78	79.6	64	63.4
Depression						
Screened positive	94	47.0	37	37.4	57	56.4
Screened negative	106	53.0	62	62.6	44	43.6
Previous or actual diagnosis of depression or anxiety disorder						
Yes	43	21.6	19	19.4	24	23.8
No	156	78.4	79	80.6	77	76.2
Experiences with other mental health training programs						
Before study entry^a^						
Yes	67	33.7	31	31.6	36	35.6
No	132	66.3	67	68.4	65	64.4
While participating^a^^,^ ^b^						
Yes	35	26.1	10	18.9	25	30.9
No	99	23.9	43	81.1	56	69.1
Experience with mindfulness-based interventions						
Yes	146	73.4	74	75.5	72	71.3
No	53	26.6	24	24.5	29	28.7
Regular mindfulness practice						
Yes	78	39.2	41	41.8	37	36.6
No	121	60.8	57	58.2	64	63.4

a Within the last 4 weeks.

b *n* = 134.

**Table 4:** Means and standard deviations for primary and secondary outcomes.

Outcome	T1				T2				6-MFU			
	Namah		Work-book		Namah		Work-book		Namah		Work-book	
	*M*	*SD*	*M*	*SD*	*M*	*SD*	*M*	*SD*	*M*	*SD*	*M*	*SD*
Primary												
Perceived stress	20.53	5.03	22.11	5.46	16.83	5.81	17.72	6.65	15.28	6.12	16.96	7.00
Secondary												
Depression	15.53	7.00	17.80	7.43	11.81	7.76	13.28	8.30	10.37	7.23	13.81	8.73
Risk factors												
Inadequate self^a^	22.81	6.82	23.76	7.45	19.25	7.42	18.88	8.24	15.56	10.63	17.56	7.63
Negative emotions	13.23	5.40	14.23	6.00	10.87	5.30	10.75	5.97	10.96	5.78	10.41	6.62
Resources												
Self-compassion	32.40	7.99	30.73	8.05	39.65	8.99	37.81	9.44	39.97	13.82	39.15	9.63
Reassured self^a^	14.83	5.83	13.66	6.15	18.56	6.70	16.91	6.70	20.06	8.47	18.18	6.84
Mindfulness	51.76	11.74	52.43	12.15	56.01	11.69	56.60	12.11	58.93	14.28	58.13	11.9
Positive Emotions	20.36	6.44	17.89	6.32	22.03	7.07	21.79	7.44	24.13	9.01	22.63	7.89
Well-being												
Autonomy^b^	10.89	2.95	10.99	3.06	12.10	2.41	12.07	2.95	13.22	3.25	12.59	2.8
Mastery^b^	11.51	2.88	10.87	2.83	12.84	3.10	12.33	2.95	13.08	3.55	12.45	2.96
Purpose^b^	11.15	2.09	11.27	2.36	10.93	2.64	11.18	2.50	10.43	1.88	11.42	2.29
Personal growth^b^	14.83	2.39	14.78	2.22	15.05	2.07	15.04	2.88	14.85	2.32	14.75	2.8
Self-acceptance^b^	12.95	2.88	12.42	3.06	13.48	2.76	13.03	3.01	13.60	3.40	13.44	2.61
Positive relations^b^	12.73	3.59	12.79	3.20	13.31	3.11	13.18	2.90	13.33	3.50	13.22	3.11
Subjective well-being	10.64	4.16	9.30	4.11	11.81	4.68	11.97	4.64	13.93	5.77	12.10	5.61
Satisfaction with life	21.97	6.17	21.16	6.58	23.40	6.23	22.39	6.59	24.57	7.95	23.74	6.34

a Subscale of forms of self-criticizing/attacking and self-reassuring scale.

b Subscale of psychological well-being scale.

*N* = 199.

### 3.3 Missing data

Baseline data were assessed for all participants. To account for missing data, multiple imputations using 20 datasets were performed (73). Primary outcome data was missing for 29.55% post-intervention and 43.96% at 6-MFU. Following Rubin's Rule (67), Table 4 shows marginal means and standard deviations at all assessment points.

### 3.4 Primary outcome

Table 5 summarizes the results at post-intervention of the respective ANCOVAs. For the primary analysis, eight weeks after randomization, perceived stress scores between the Namah and workbook group did not differ significantly [*F*_1,186.2_ = 0.60; *p* = 0.44; *d* = 0.13 (*95%-CI* = −0.14, 0.41)]. Likewise, no significant differences in perceived stress were observed between the training formats at 6-month follow-up [*F*_1,188.7_ = 1.76; *p* = 0.19; *d* = 0.14 (*95%-CI* = −0.17, 0.45)].

**Table 5:** Results of ANCOVAs for primary and secondary outcomes at T2 and T3.

Outcome	Differences between study conditions (T2)		Differences between study conditions (T3)	
	ANCOVA (*F_186.2_*)	Cohen's *d* [95% *CI*]^a^	ANCOVA (*F_188.7_*)	Cohen's *d* [95% *CI*]^a^
Primary				
Perceived stress	0.60	0.13^ns^ [−0.14, 0.41]	1.76	0.14^ns^ [−0.17, 0.45]
Secondary				
Depression	0.75	0.13^ns^ [−0.15, 0.41]	1.91	0.23^ns^ [−0.05, 0.58]
Risk factors				
Inadequate self^b^	0.29	0.04^ns^ [−0.32, 0.24]	1.21	0.18^ns^ [−0.26, 0.63]
Negative emotions	0.26	0.03^ns^ [−0.31, 0.25]	0.56	0.13^ns^ [−0.14, 0.39]
Resources				
Self-compassion	0.72	0.13^ns^ [−0.14, 0.41]	0.28	0.02^ns^ [−0.49, 0.52]
Reassured self^b^	0.76	0.14^ns^ [−0.14, 0.42]	1.69	0.17^ns^ [−0.19, 0.53]
Mindfulness	1.38	0.05^ns^ [−0.23, 0.33]	0.65	0.14^ns^ [−0.56, 0.83]
Positive emotions	0.25	0.02^ns^ [−0.30, 0.25]	0.79	0.03^ns^ [−0.39, 0.46]
Well-being				
Autonomy^c^	0.30	0.03^ns^ [−0.25, 0.31]	1.24	0.09^ns^ [−0.05, 0.23]
Mastery^c^	1.23	0.18^ns^ [−0.10, 0.46]	1.59	0.04^ns^ [−0.16, 0.23]
Purpose^c^	0.50	0.09^ns^ [−0.18, 0.36]	3.30	0.12^ns^ [−0.19, 0.22]
Personal growth^c^	0.16	0.004^ns^ [−0.27, 0.28]	0.48	0.01^ns^ [−0.10, 0.12]
Self-acceptance^c^	1.20	0.14^ns^ [−0.13, 0.42]	1.04	0.01^ns^ [−0.17, 0.20]
Positive relations^c^	0.29	0.02^ns^ [−0.25, 0.30]	0.44	0.02^ns^ [−0.17, 0.20]
Subjective well-being	0.19	0.01^ns^ [−0.27, 0.29]	3.04	0.15^ns^ [−0.11, 0.41]
Satisfaction with life	1.45	0.14^ns^ [−0.13, 0.42]	0.75	0.04^ns^ [−0.25, 0.33]

a Cohen's *d* was calculated by using pooled standard deviation.

b Subscale forms of self-criticizing/attacking and self-reassuring scale.

c Subscale psychological well-being scale.

*N* = 199.

ns *p* > .05.

Statistically-significant reductions in perceived stress between pre- and post-intervention were identified within both groups [Namah group_pre−post_: *t*_1,118.7_ = 3.74; *p* ≤ .001; *d* = 0.68 (*95%-CI* = 0.39, 0.97); workbook group_pre−post_: *t*_1,118.7_ = 4.79; *p* ≤ .001; *d* = 0.79 (*95%-CI* = 0.50, 1.08)]. This represents a percentage of stress reduction of 18.02% in the Namah and of 19.86% in the workbook group on average. Within-group analyses comparing pre-intervention and 6-MFU also revealed statistically-significant reductions in both groups [Namah group_pre−6−MFU_: *t*_1,74.4_ = 3.95; *p* ≤ .001; *d* = 0.62 (*95%-CI* = 0.34, 0.91); workbook group_pre−6−MFU_: *t*_1,74.4_ = 4.00; *p* ≤ .001; *d* = 0.57 (*95%-CI* = 0.28, 0.85)]. The percentages of stress reduction observed in the Namah and workbook groups, relative to baseline, were 25.57% and 23.29%, respectively (see Supplementary Materials 2, 3).

### 3.5 Secondary outcomes

All between-group differences in secondary outcomes, both post-intervention and at 6-MFU, were non-significant (Table 5).

Concordantly, there was a reduction in symptoms of depression in both groups from baseline to post-intervention [Namah *d* = 0.30 (*95%-CI* = 0.02, 0.58); workbook *d* = 0.48 (*95%-CI* = 0.20, 0.76)] that was maintained after six months [Namah *d* = 0.33 (*95%-CI* = 0.05, 0.61); workbook *d* = 0.25 (*95%-CI* = −0.03, 0.53)]. This corresponds to reductions in symptom severity of 24% in the Namah and 25% in the workbook group, that were maintained at six months (Namah = 33%; workbook = 22%). Likewise, short-term and longer-term positive developments were observed in both groups for fostering self-compassion and a reassured self, and for decreasing inadequate self, representing outcomes directly related to the content of the self-compassion interventions. However, we did not observe any changes in the more general and positive indicators of mental health, including life satisfaction, or in facets of psychological well-being, like personal growth, the one exception being subjective well-being after six months, for which both groups reported positive developments (see Supplementary Materials 2, 3).

### 3.6 Sensitivity analysis

As baseline differences in depressive symptom severity were observed between groups, the baseline CES-D score was included as a covariate in the primary ANCOVA, but this addition did not yield any statistically-significant effect (*F*_1,161.91_ = 0.61; *p* = 0.43). Results for the primary outcome obtained with the study completer sample [post-intervention: *F*_1,137_ = 0.13, *p* = 0.72, *d* = 0.12 (*95%-CI* = −0.22, 0.46); 6-MFU: *F*_1,112_ = 1.42, *p* = 0.24, *d* = 0.32 (*95%-CI* = −0.07, 0.71)] were in accordance with those in the ITT sample, showing no statistically significant between-group effect. Analyses employing the intervention completer sample were similar [post-intervention: *F*_1,97_ = 0.10, *p* = 0.77, *d* = 0.17 (*95%-CI* = −0.44, 0.78); 6-MFU: *F*_1,113_ = 1.63, *p* = 0.21, *d* = 0.35 (*95%-CI* = −0.11, 0.82)]. Further sensitivity analysis using a mixed model revealed no statistically-significant interaction between time and intervention (post-intervention: *β* = 0.54, *SE* = 1.05, *p* = 0.61; 6-MFU: *β* = −0.31, *SE* = 1.17, *p* = 0.79).

#### 3.6.1 Co-interventions

During the study period, 30.9% of participants practicing with the workbook reported having also used other similar mental health training programs, vs. 18.9% of participants training with the Namah program. In addition, 36.6% vs. 41.8% in the workbook and Namah groups claimed regularly practicing mindfulness-based interventions, respectively. Neither appeared to have any significant effect on the results as covariates.

#### 3.6.2 Adherence

Adherence to the Namah intervention was assessed based on session completion data automatically logged by the digital platform. On average, participants in the Namah group completed three sessions by the post-assessment and four sessions by 6-MFU. 14.29% attended at least six out of the seven sessions by eight weeks and 30.61% by 6-MFU. There was no indication of any dose-response relationship between the number of completed modules and level of stress post-intervention, (*r* = −.04; *p* = .79). For the workbook group no adherence data were collected. However, significantly more participants in the Namah group reported still practicing the training exercises in their everyday life (*t* = 2.12; *p* = .04) or having changed their habits (*t* = 2.50; *p* = .02) than participants in the workbook group at 6-MFU after the intervention period.

### 3.7 Reliable improvement and deterioration rates

Concerning the primary outcome of perceived stress, 53.06% in the Namah group and 59.41% in the workbook group reported reliable improvement between pre- and post-intervention. Corresponding deterioration rates ranged from 10.89% in the workbook group to 15.31% in the Namah group. By 6-MFU, 59.18% in the Namah and 67.33% in the workbook group reported reliable improvement. Whereas deterioration was experienced by 8.16% in the Namah and 12.87% in the workbook group at 6-MFU.

Similar patterns arose regarding the secondary outcome depression with 40.82% of participants in the Namah group and 53.47% in the workbook group reporting reliable improvement post-intervention. Whereas at 6-MFU, 54.08% of participants in the Namah group and 51.49% in the workbook group reported reliable improvement. Deterioration occurred post-intervention for 13.27% of participants in the Namah group and 14.85% participants trained with the workbook. At 6-MFU, deterioration was reported by 11.22% participants of Namah and 18.81% of the workbook group.

### 3.8 Client satisfaction

Overall, 67.4% of participants (*n* = 134) answered the client satisfaction questionnaire. Participants in the Namah training program were significantly more satisfied with the intervention (total score: *M* = 28.08, *SD* = 4.71) than who trained with the workbook (total score: *M* = 22.07; *SD* = 6.27; *t* = 6.32, *p* < .001). In the Namah group, 96.2% of participants claimed being either partly or entirely satisfied with the intervention, while 94.3% reported they would recommend the intervention to a friend. In comparison, 70.4% in the workbook group reported being partly or entirely satisfied and 65.4% claimed they would recommend it to a friend (Table 6).

**Table 6:** Descriptive results for the CSQ-8 scale.

CSQ-8 Item^a^	Namah Intervention				Workbook			
	Agreement^b^				Agreement^b^			
	*M*	*SD*	*n*	*%*	*M*	*SD*	*n*	*%*
Quality of the training	3.68	0.58	50	94.34	3.01	0.77	66	81.48
Received the training I wanted	3.47	0.70	49	92.45	2.63	0.93	46	56.79
Training met my needs	3.43	0.69	49	92.45	2.64	0.93	51	62.96
Would recommend the training to a friend	3.68	0.64	50	94.34	2.74	1.01	53	65.43
Received the amount of help I wanted	3.45	0.75	49	92.45	2.63	0.87	48	59.26
Training helped me to deal more effectively with my problems	3.28	0.79	46	86.79	2.79	0.85	58	71.60
Generally satisfied with the training	3.58	0.63	51	96.23	2.84	0.90	57	70.37
Would use the training again	3.49	0.80	47	88.68	2.79	1.05	53	65.43
Total score	28.08	4.71			22.07	6.27		

a Not at all (1), Rather not (2), Yes in parts (3), Yes totally true (4).

b Responded with ≥3; n_Namah_ = 53. n_workbook_ = 81.

## 4 Discussion

The present study investigated the effectiveness of a digital and a bibliotherapeutic self-compassion intervention at reducing stress in the general population. Both low-threshold interventions had a significant and meaningful effect on stress-reduction from pre- to post-intervention, which was maintained through six months. More than half the participants in both self-compassion intervention groups reported a reliable improvement in perceived stress. Of note, however, on head-to-head comparison, no significant differences were observed between the guided digital intervention Namah and an existing, broadly-available self-help workbook. For secondary outcomes that include depressive symptoms and various risk-factors, as well as resources, findings showed a similar pattern of significant within-group but absent between-group effects. However, participants in the guided Namah group reported higher levels of satisfaction with the intervention and were more likely to integrate the learned self-compassion exercises into their everyday lives after the intervention period.

A major, yet unexpected result was that both self-compassion interventions were equally effective at reducing stress and symptoms of depression, and at fostering self-compassion. Compared with benchmarks (95, 96) using anchor methods (97) to define a clinically-important reduction in depression severity, changes between 22% and 33% indicate practically important effects in participants with mild to moderate symptomatology at baseline. Within-group effects in both groups ranged from *d* = 0.30 to *d* = 0.48 for depression, and thereby exceeded the benchmark for solely time-dependent changes in depressive symptoms that were found to be *d* = 0.12 in the meta-analyses published by Tong et al. (74), relative to untreated controls, in RCTs of samples with mild symptom severity. Unfortunately, such benchmarks are not yet available for perceived stress, our primary outcome. However, Sommers-Spijkerman et al. (38) reported for a compassion workbook augmented with email guidance a reduction in perceived stress within the intervention group of about 20%, which also was found in the present study. Regarding rates for reliable improvement in perceived stress post-intervention, the percentage of improvement in the present study (Namah = 53.06%; workbook = 59.41%) were similar to previously-reported rates for a guided digital stress-management intervention (61.40%) when compared to 25.0% in the waitlist control group (75). Regarding effects on stress and depression, Nixon et al. (76) found that effects on depression are mediated by effects on stress, suggesting that stress is not only an etiological risk-factor, but also a mechanism of change.

Although results from a prior meta-analysis (10) suggest that guided interventions are associated with stronger effects on stress than unguided interventions, we did not observe any significant differences between the two intervention groups. This pattern of results was confirmed in several sensitivity analyses. Nevertheless, at a descriptive level, six months after randomization Namah group participants reported less stress and symptoms of depression than the workbook group, but these differences in the expected direction were not statistically-significant, small in size and therefore it is more conservative to conclude that no differences exist. Regarding digital self-compassion interventions, a recent meta-analysis by Han and Kim (28) showed no superiority when compared to other interventions to reduce stress, which is in line with the present results.

Few studies have compared digital interventions and bibliotherapy directly. Smith et al. (36) found that guided internet-delivered cognitive behavior therapy yielded similar effects as self-help bibliotherapy, both being effective at reducing symptoms of depression. Similarly, Hedman and colleagues (37) found within-group effects on various mental health outcomes for both guided and unguided digital interventions and self-help bibliotherapy, but no between-group effects. Furthermore, preliminary results have suggested that a self-compassion workbook is as effective at reducing trauma related distress as a stress inoculation workbook (77). The stronger-than-expected effectiveness of the bibliotherapeutic intervention might be explained by the step-by-step mode of deliverance. Smith et al. (36) advised participants to work through one chapter per week and Hedman et al. (37) had weekly contact via email for assessments, both with a timeframe of about 12 weeks. Similarly, participants in the current study's workbook group were contacted seven times, corresponding to the number of sessions in the Namah group, for providing weekly questions about their well-being and self-care. This regular contact may have acted as a form of supportive accompaniment and potentially contributed to the observed effects. Overall, effective bibliotherapy to promote self-compassion might require more than just providing self-help materials. A clearly-communicated timeframe during which participants work with the materials, a weekly rhythm, the inclusion of practical exercises beyond mere information, and a minimum level of personal contact appear to be beneficial conditions as demonstrated by the feasible and effective approach of Sommers-Spijkerman et al. (38) in the context of public mental health.

Self-compassion interventions are sometimes labeled “positive” [e.g., (78)], which can lead to the implicit assumption that all their effects are positive and beneficial for participants. Another important observation in the present trial is the substantial rate of participants reporting reliable deterioration in both groups, slightly over 10%. Recently, similar findings were reported for another positive intervention, namely a gratitude training program (44). While the personal support in the present study seemed not to enhance the programs' effectiveness, it can still be considered a safety measure for those experiencing worsening [cf., (34)].

Regarding personal support, another aspect to consider is the greater satisfaction of participants training with the guided self-compassion compared to the self-help intervention, which is consistent with results showing greater acceptance of guided interventions in the general population (79). Personal support has also been found to increase adherence to interventions aiming to reduce stress (80).

Finally, we found both interventions to be effective at promoting self-compassion, but not for more general and positive indicators of mental health. There are various explanations for this. First, meta-analytic findings have revealed positive effects of self-compassion interventions on various facets of self-compassion (27), indicating that self-compassion can be cultivated. Second, our findings suggest that both interventions managed to reach their core goals by promoting self-kindness, recognizing the imperfection of humans and contributing to more balanced perspectives [cf., (53)]. Moreover, participants perceived themselves to be less inadequate; for example, becoming less disappointed in and more able to reassure themselves [e.g., by forgiving themselves (49)]. Pre-intervention scores for inadequate-self and reassured-self were close to levels in clinical samples (49). Post-intervention scores reached roughly the levels of the non-clinical population, indicating meaningful effects. Third, no consistent effects on life satisfaction or psychological well-being were found. Among the many outcomes investigated by Ferrari et al. (27), they identified the smallest effects for life satisfaction. One possible explanation may be found in the intensity of the intervention relative to its breadth and range. Longer and more intensive interventions may be needed to see positive spillover effects from more specific characteristics, such as stress and self-compassion, to more general measures that take someone's entire life situation into account.

### 4.1 Strengths and limitations

To the best of our knowledge, this is the first trial comparing two low-threshold self-compassion interventions in the general population. The choice of an existing, broadly-available workbook ensured that the control intervention is of practical importance [cf., (81)]. While most studies on self-compassion interventions employed symptomatic inclusion criteria, referring to an indicated prevention setting (28) or were conducted in clinical samples (32), we reduced inclusion and exclusion criteria to a minimum to mimic evaluation under real-life conditions from the perspective of universal prevention, thereby enhancing generalizability. The study addresses several issues that were identified by Ferrari et al. (27) as limitations in interventional self-compassion research: the study was registered prior to its initiation; the follow-up period of six months allows conclusions beyond the immediate post-intervention effects; and results are reported according to CONSORT guidelines. Methodically, a variety of sensitivity analyses, including mixed modelling (68), study and intervention completer analyses, and considering baseline differences and co-interventions by applying ANCOVA (69–71) all led to the same conclusions, strengthening confidence in the results. Finally, the study adds to the rapidly-growing, yet heterogeneous body of evidence supporting the feasibility and efficacy of digital self-compassion interventions (28).

Although the present findings are promising overall, it remains important to carefully consider several limitations. First, based on previous findings on the effectiveness of similarly-designed digital mental health interventions [e.g., (82)], the current trial was designed as a superiority study and non-significant differences are not proof of either equivalence or non-inferiority (83) of self-help bibliotherapy compared to the guided digital intervention.

Second, including a third experimental group that did not receive any intervention would have strengthened the internal validity regarding conclusions about the effectiveness of the two interventions. This said, since published meta-analytic evidence supports the efficacy of self-compassion interventions compared to no intervention, the ethical question arises as to whether withholding effective interventions is justified or whether this conflicts with the principles of beneficence and non-maleficence [cf., (84, 85)].

Third, participants in the workbook group were more frequently engaged in other mental health interventions during the study period compared to those in the Namah group. Although the differences using co-interventions during the trial period did not appear to have a significant effect as covariates, this has several implications. It seems crucial to assess reasons for and frequency of uptake of co-interventions.

Fourth, as typical in mental health intervention studies (86), around 80% of participants in the current study were higher-educated, middle-aged and female; therefore, caution is warranted when generalizing the findings to less-educated and male populations. As indicated by the present trial's uptake rates, self-compassion interventions appear to appeal most to well-educated women. One explanation might be found in meta-analytic conclusions that women are less self-compassionate (87) and probably more self-critical, thus, potentially making the self-compassion interventions more appealing to them. Therefore, women may be more willing to participate in a self-compassion program. For men, participation in such a training program might be less attractive, which may be indicated by results from Gilbert et al. (88) who found that men tend to show greater fears and resistances to compassion which may reduce their likelihood of engaging in such interventions.

Fifth, given that 56.04% of participants provided data at six-month follow-up, these results should be interpreted with caution, despite using multiple imputations, as recommended by Graham et al. (73), to adjust for missing data and corroborating results through various forms of sensitivity analysis, including mixed modelling.

Sixth, it should be acknowledged that there are multiple approaches to (self-)compassion training. For example, Compassion Focused Therapy (CFT), as proposed by Gilbert (98), may be regarded as broader, as it considers three types of compassion: compassion for others, compassion received from others, and self-compassion. While each of the three can be a focal point in CFT, the Namah intervention focuses on self-compassion. Furthermore, Gilbert's interventional approach includes challenging the fear of self-compassion and the fear of compassion from others, while Namah does not consider the idea that individuals may experience fear of compassion. Finally, working on self-criticism is equally vital in both CFT and Namah, shame is additionally addressed in CFT as a major transdiagnostic factor.

Finally, it remains to be stated that with only 14% of intervention completer, adherence rates for Namah were comparably low (80). Reasons for the low level of adherence may relate to the intervention's design and/or technical issues, especially for the mobile component. Although the Namah intervention was pilot tested before the study began without any reported problems or inconveniences, improvements to the design (e.g., session length) might have reduced the dropout rate. Another reason for the low adherence may be the possibility that individuals can benefit from the intervention without completing the protocol (89). Lutz et al. (90) has found that a substantial proportion of participants improve early, even after registering and screening for study participation. In the present study, no dose-response relationship was observed. This means that, on average, participants who completed more sessions did not show greater improvement than those who completed fewer sessions. Similar results were also reported by Krieger et al. (82). However, qualitative research is needed to understand the reasons for early termination and to identify ways to improve the intervention. It should also be noted that adherence data were available solely for the digital training group, limiting the evaluation of a potential dose–response relationship to participants of the Namah intervention.

### 4.2 Future directions

First, the present study demonstrated that self-compassion interventions could be an effective component and enrich the range of interventions as part of an overall strategy for mental health promotion in the general population [cf., (38)]. Since other, longer-established interventions exist that also produce substantial effects on stress or depressive symptomatology—like stress management training programs (10), mindfulness-based interventions (91), and depression prevention interventions (92)—research is needed to determine if increasing the diversity of interventional approaches will lead to greater overall reach with evidence-based interventions in an increasingly-diverse population.

Second, while there was great interest in bibliotherapy in the past (34, 35), this effective approach appears to have fallen out of fashion with the rise of digital interventions. Results of the present study—along with those reported by Smith et al. (36) and Hedman et al. (37)—suggest, however, that the value of printed material (e.g., books) might currently be underestimated. Moreover, digital technology could be used as a facilitator for traditional bibliotherapy, for example, by providing weekly questions for reflection, monitoring progress, sending reminders or reinforcing massages, and facilitating contact with mental health experts in cases of deterioration.

Third, to date, a variety of self-compassion interventions have been developed and positively evaluated, comparing them to non-intervention controls (28). For progress in our understanding, and thereby optimize and identify the most effective self-compassion interventions, it is vital that promising, existing effective interventions be used as comparators in future studies, as noted in Krieger et al. (82). This is also requested in research ethics guidelines (85).

Finally, after establishing the efficacy and effectiveness of self-compassion interventions, a warranted next step will be to examine the underlying key mechanisms through which they exert their effects (27). Holmes et al. (93) proposed that understanding mechanisms is a pathway to refining interventions by focusing on their essential components. As proposed by Cha et al. (94), a number of behavioral, motivational, and physiological processes may serve as candidate mechanisms.

### 4.3 Conclusion

Despite the limitations and further research avenues that remain, the present study suggests that both digital and traditional bibliotherapeutic self-compassion interventions may be effective at promoting more compassionate attitudes towards oneself in the general population. Reduced levels of stress were found after both self-compassion interventions, that is of importance as stress is a major risk factor for a variety of physical and mental disorders, including depression. Likewise, reduced symptoms of depression were observed for both interventions. The availability of an eCoach seems to be associated with higher intervention satisfaction, but may also serve as a safety measure in cases of deterioration. Over the past decade, bibliotherapy's effectiveness appears to have been underestimated, but it may hold considerable potential in the context of universal prevention—particularly when delivered in a structured manner, supported by personal contact, and potentially enhanced through digital applications that facilitate these success factors.

## Data Availability

The raw data supporting the conclusions of this article will be made available by the authors, without undue reservation.
