# Supplementary material for: A two-armed pragmatic randomized controlled trial comparing the effectiveness of two self-compassion interventions at reducing perceived stress
Source: Front Digit Health. 2026 Feb 9;8:1680033. doi: 10.3389/fdgth.2026.1680033 (PMC12926457; doi:10.3389/fdgth.2026.1680033)
Supplement: Supplementary file 1 [file Datasheet1.pdf]

Supplementary material

Content

S1 Occupational health outcomes

S1.1 Measures

S1.2 Means and standard deviations

S1.3 Differences between study conditions and within groups at T2

S1.4 Differences between study conditions and within groups at T3

S2 Results for primary and secondary outcomes within-group at T2

S3 Results for primary and secondary outcomes within-group at T3

S4 Overview: Content of the Namah intervention

S5 Overview: Exercises to strengthen self-kindness, common humanity and mindfulness

S6 Overview: Content of the workbook

S1. Occupational health outcomes

S1.1 Measures

Irritation was measured using the Irritations Scale (IS; Mohr et al., 2006; α_{total}=.89) consisting of eight Items and two subscales (emotional, cognitive irritation; sum range: 5-35 for emotional irritation and 3-21 for cognitive irritation).

The Recovery Experience Questionnaire (RECQ, Sonnentag and Fritz, 2007; α=.85), which has 16 Items and four subscales (Detachment, Recovery, Mastery, Control), was used to assess recovery (sum range: 4-20 per subscale).

As a measurement of self-efficacy, the Short Occupational Self-Efficacy Scale (OSSF, Rigotti et al., 2008; α_{total}=.87), with six Items and a sum range of 6 to 36, was used.

The Effort-Reward-Imbalance Scale (ERI; Siegrist et al., 2009; α=.74-.79), which has 10 Items and two subscales (effort, reward), was used as a possible control variable (sum range: 3-12 for the effort scale; 7-28 for the reward scale).

S1.2. Means and standard deviations of occupational health outcomes

Outcome	T1				T2				6-MFU			
	Namah		Work-book		Namah		Work-book		Namah		Work-book	
	M	*SD*	*M*	*SD*	*M*	*SD*	*M*	*SD*	*M*	*SD*	*M*	*SD*
Emotional irritation	19.95	6.71	19.79	6.69	18.90	9.05	16.96	6.40	15.47	8.59	16.85	8.16
Cognitive irritation	12.41	4.47	14.08	4.47	10.84	5.62	12.27	4.58	10.41	5.21	11.22	5.67
Self-efficacy	24.06	5.01	23.70	4.88	24.17	6.51	25.47	5.85	26.74	6.83	26.32	5.96
Job-stress recovery	49.27	9.35	47.43	8.75	52.32	10.65	51.47	10.39	54.76	11.00	52.96	12.83

S1.3 Differences between study conditions and within-group comparisons for occupational health outcomes (T2)

	Differences between study conditions		Differences within group Namah		Differences within group Workbook	
Outcome	ANCOVA $F_{24.9}$	Cohen's *d* [95% *CI*][a]	$t_{22.8}$	Cohen's *d* [95% *CI*][a]	$t_{24.4}$	Cohen's *d* [95% *CI*][a]
Emotional irritation	1.27	0.25^{ns} [-0.03, 0.53]	0.37	0.05^{ns}[-0.23, 0.33]	1.68	0.24^{ns} [-0.04, 0.52]
Cognitive irritation	1.26	0.02^{ns} [-0.26, 0.30]	0.03	0.01^{ns}[-0.27, 0.29]	0.92	0.19^{ns} [-0.09, 0.47]
Self-efficacy	1.45	0.17^{ns} [-0.11, 0.45]	0.23	0.04^{ns}[-0.24, 0.32]	0.33	0.06^{ns} [-0.22, 0.34]
Job-stress recovery	1.67	0.04^{ns} [-0.23, 0.32]	0.49	0.05^{ns}[-0.23, 0.33]	2.55	0.25* [-0.03, 0.53]

Note. [a] Cohen's *d* was calculated by using pooled standard deviation. ^{ns} $p > .05$; * $p \leq .05$; ** $p \leq .01$; *** $p \leq .001$.

S1.4 Differences between study conditions and within group comparisons for occupational health outcomes (T3)

	Differences between study conditions		Differences within group Namah		Differences within group Workbook	
Outcome	ANCOVA $F_{24.6}$	Cohen's *d* [95% *CI*][a]	$t_{31.6}$	Cohen's *d* [95% *CI*][a]	$t_{39.7}$	Cohen's *d* [95% *CI*][a]
Emotional irritation	1.05	0.19^{ns} [-1.07, 1.45]	1.57	0.20 ^{ns} [-0.08, 0.48]	1.07	0.13^{ns} [-0.15, 0.40]
Cognitive irritation	2.02	0.05^{ns} [-1.50, 1.60]	0.79	0.15 ^{ns} [-0.12, 0.43]	1.62	0.28 ^{ns} [-0.01, 0.57]
Self-efficacy	1.14	0.04^{ns} [-0.80, 0.89]	1.13	0.18 ^{ns} [-0.10, 0.45]	1.86	0.29 ^{ns} [0.01, 0.57]
Job-stress recovery	2.12	0.06^{ns} [-2.02, 2.15]	1.42	0.13 ^{ns} [-0.14, 0.41]	1.12	0.09 ^{ns} [-0.18, 0.37]

Note. [a] Cohen's *d* was calculated by using pooled standard deviation. ^{ns} $p > .05$; * $p \leq .05$; ** $p \leq .01$; *** $p \leq .001$.

S2 Results for primary and secondary outcomes within-group at T2

	Differences within group Namah		Differences within group Workbook	
Outcome	$t_{118.7}$	Cohen's *d* [95% *CI*][a]	$t_{118.7}$	Cohen's *d* [95% *CI*][a]
**Primary**				
Perceived stress	3.74	0.68***[0.39, 0.97]	4.79	0.79***[0.50, 1.08]
**Secondary**				
Depression	2.29	0.30*[0.02, 0.58]	3.81	0.48***[0.20, 0.76]
**Risk factors**				
Inadequate self[b]	2.83	0.40**[0.12, 0.68]	4.29	0.55***[0.27, 0.83]
Negative emotions	2.58	0.49**[0.21, 0.77]	3.94	0.66***[0.37, 0.94]
**Resources**				
Self-compassion	4.60	0.54***[0.25, 0.82]	5.57	0.63***[0.34, 0.91]
Reassured self[b]	3.07	0.50**[0.22, 0.78]	3.63	0.57***[0.29, 0.85]
Mindfulness	0.63	0.05[ns][-0.23, 0.33]	2.28	0.19*[-0.09, 0.47]
Positive emotions	1.49	0.22[ns][-0.06, 0.50]	3.98	0.58***[0.30, 0.86]
**Well-being**				
Autonomy[c]	2.81	0.80**[0.51, 1.08]	2.40	0.80*[0.51, 1.09]
Mastery[c]	2.82	0.96**[0.67, 1.25]	3.51	1.20***[0.90, 1.50]
Purpose[c]	0.60	0.25[ns][-0.03, 0.53]	0.31	0.13[ns][-0.15, 0.41]
Personal growth[c]	0.66	0.30[ns][0.02, 0.58]	0.71	0.27[ns][-0.01, 0.55]
Self-acceptance[c]	1.18	0.43[ns][0.15, 0.71]	1.47	0.49[ns][0.21, 0.77]
Positive relations[c]	1.04	0.31[ns][0.03, 0.59]	0.97	0.32[ns][0.04, 0.60]
Subjective well-being	1.75	0.40[ns][0.12, 0.68]	3.80	0.86***[0.57, 1.15]
Satisfaction with life	1.34	0.22[ns][-0.06, 0.50]	1.25	0.19[ns][-0.09, 0.47]

Note. [a] Cohen's *d* was calculated by using pooled standard deviation. [b] Subscale of Forms of Self-Criticizing/Attacking and Self-Reassuring Scale. [c] Subscale of Psychological Well-being Scale. $N = 199$. [ns] $p > .05$; * $p \leq .05$; ** $p \leq .01$; *** $p \leq .001$.

S3 Results for primary and secondary outcomes within-group at T3

	Differences within group Namah		Differences within group Workbook	
Outcome	$t_{74.4}$	Cohen's *d* [95% *CI*][a]	$t_{74.4}$	Cohen's *d* [95% *CI*][a]
**Primary**				
Perceived stress	3.95	0.62***[0.34, 0.91]	4.00	0.57***[0.28, 0.85]
**Secondary**				
Depression	2.53	0.33**[0.05, 0.61]	2.30	0.25*[-0.03, 0.53]
**Risk factors**				
Inadequate self[a]	3.24	0.34**[0.06, 0.62]	3.65	0.43***[0.14, 0.71]
Negative emotions	1.23	0.19[ns][-0.08, 0.47]	2.67	0.37**[0.09, 0.65]
**Resources**				
Self-compassion	3.29	0.29**[0.01, 0.56]	4.20	0.41***[0.13, 0.69]
Reassured self[a]	3.22	0.42**[0.13, 0.70]	3.07	0.42**[0.14, 0.70]
Mindfulness	2.40	0.16*[-0.11, 0.44]	2.33	0.17*[-0.11, 0.45]
Positive emotions	1.94	0.22[+][-0.06, 0.50]	3.46	0.42***[0.14, 0.70]
Autonomy[b]	1.47	0.41 [ns][0.12, 0.69]	2.35	0.70*[0.41, 0.98]
Mastery[b]	1.66	0.46 [ns][0.18, 0.74]	2.62	0.79**[0.50, 1.08]
Purpose[b]	1.24	0.54 [ns][0.26, 0.83]	0.54	0.21 [ns][-0.07, 0.48]
Personal growth[b]	0.99	0.35 [ns][0.07, 0.63]	1.20	0.40 [ns][0.12, 0.69]
Self-acceptance[b]	0.01	0.01 [ns][-0.27, 0.28]	1.72	0.55 [ns][0.26, 0.83]
Positive relations[b]	0.24	0.06 [ns][-0.22, 0.33]	0.36	0.10 [ns][-0.17, 0.38]
Subjective well-being	2.30	0.41*[0.13, 0.69]	2.95	0.53**[0.25, 0.81]
Satisfaction with life	0.44	0.06 [ns][-0.22, 0.33]	1.79	0.25 [ns][-0.03, 0.53]

Note. [a] Subscale of Forms of Self-Criticizing/Attacking and Self-Reassuring Scale. [b] Subscale of Psychological Well-being Scale. [ns] $p > .05$; + $p \leq .1$; * $p \leq .05$; ** $p \leq .01$; *** $p \leq .001$.

S4 Overview: Content of the Namah intervention

Session topic	Objectives	Exercises
Becoming aware of one's own self-criticism	To become acquainted with the training program and the concept of self-compassion, and become aware when criticizing oneself	• Situation analysis of self-critical behavior guided by audio-sequences<br>• Guided meditation on individual objectives within the training<br>• Psychoeducational elements on the vicious circle of self-criticism and the concept of self-compassion by video
Developing a friendly approach to oneself	To draw attention to both the functionality and dysfunctionality of self-criticism, and encourage participants to adopt a self-compassionate perspective	• Advantages and disadvantages (functionality) of self-criticism<br>• Guided meditation on one's current state of self-compassion
Perceiving self mindfully	To learn how a mindful attitude towards oneself helps to strengthen self-compassion	• Psychoeducational elements (e.g., videos) on mindfulness and how it is connected with self-compassion<br>• Quiz for non-judgmental descriptions and own experiences<br>• Guided breathing meditation<br>• Integrating mindfulness into everyday life
Building a helpful mindset	To learn how to identify self-critical thoughts and convert them into constructive thoughts	• Identifying self-critical thoughts<br>• Expressing constructive self-criticism by audio-exercise<br>• Consolidating helpful thoughts
Learning to deal with difficult emotions	To cope with difficult emotions and reflect on their appropriateness	• How guilt and shame manifest and what functions they serve<br>• Situation analysis on shame and guilt and the appropriateness of feelings<br>• Behavioral approaches to deal with shame and guilt
Developing self-supportive behavior	To determine what one feels good about	• Generating ideas for self-supporting activities<br>• Planning schedule for self-supporting activities
Being a friend to oneself in the future	To transfer what has been learnt so far into the future and build on it	• Dealing with setbacks<br>• Summary of previous training content and review of progress<br>• Creating a plan for further training
Optional modules available throughout the Namah training program		
Personal objectives	To set realistic objectives	• Identifying Everest goals and working out subgoals
Perfectionism	To learn to deal with perfectionist tendencies	• Identifying and questioning inner convictions
Other people's expectations	To learn how to deal with external expectations	• Reflecting external expectations and (if applicable) creating a plan to break away from them

Individual strength	To learn to recognize and use one's own strengths	• Becoming aware of and identifying one's own strengths and planning how to use them more often
Personal values in life	To discover one's own values and act in accordance with them	• Discovering one's values and figuring out how to live them

S5 Overview: Exercises, that were supposed to strengthen self-kindness, common humanity and mindfulness

Component	Exercise Namah
Self-Kindness	• Expressing supportive and constructive self-feedback instead of harsh self-criticism and consolidating helpful thoughts (audio-exercise)<br>• Generating ideas for self-supporting activities<br>• Planning schedule for self-supporting activities
Common Humanity	• Acknowledging that everyone has both strengths and weaknesses<br>• Becoming aware of and identifying one's own strengths and planning how to use them more often<br>• A general look at the role of shame and guilt and their functions in human beings
Mindfulness	• Quiz for non-judgmental descriptions and own experiences<br>• Guided breathing meditation<br>• Integrating mindfulness into everyday life

S6 Overview: Content of the workbook

Section topic	Objectives	Exercises
Four steps towards self-empathy	To pay attention to oneself and one's own needs	• Psychoeducational input<br>• Situation analysis<br>• Reflective questions
Reflecting on different personality traits and needs	To acquire knowledge regarding the intricacies of one's own identity and the acknowledgement of the existence of diverse facets within one's personality	• Introducing the inner family<br>• Analysis of one's own internal components and derivation of underlying needs<br>• Tips on acknowledging and accepting one's innermost aspects
Discovering and recognizing one's needs	To acknowledge the importance of one's personal needs	• Psychoeducational input on the functionality of needs<br>• Situation analysis<br>• Analogue exercise on how fulfilled needs may feel
Freeing one's self from unwanted habits	To recognize the need underlying each unwanted habit	• Situation analysis<br>• Psychoeducational input, examples and reflecting on questions about changing habits
Celebrating success and inner strength	To shift attention away from deficits toward strengths	• Situation analysis<br>• Guiding principles
Consolidating the five pillars of self-compassion	• To recognize oneself mindfully<br>• To take responsibility for one's own needs	• Situation analysis on mindful self-perception<br>• Thought and emotion analyses

	• To prioritize self-care activities<br>• To cultivate human dialogue<br>• To translate self-judgement into needs and feelings	• Psycho-educative elements and reflective questions<br>• Listing self-care activities that worked well in the past<br>• Expressing one's thoughts and feelings firmly and respectfully<br>• Gratitude letter<br>• Short stories, examples and writing exercise on how to turn self-judgement into needs and feelings

References

Mohr, G., Müller, A., Rigotti, T., Aycan, Z., & Tschan, F. (2006). The assessment of psychological strain in work contexts. European Journal of Psychological Assessment, 22(3), 198-206. doi: 10.1027/1015-5759.22.3.198.

Rigotti, T., Schyns, B., & Mohr, G. (2008). A short version of the occupational self-efficacy scale: Structural and construct validity across five countries. Journal of Career Assessment, 16(2), 238-255. https://doi.org/10.1177/1069072707305763

Siegrist J, Wege N, Pühlhofer F, Wahrendorf M. (2009). A short generic measure of work stress in the era of globalization: Effort-reward imbalance. Int Arch Occup Environ Health 82:1005–1013. https://doi.org/10.1007/s00420-008-0384-3

Sonnentag, S., & Fritz, C. (2007). The Recovery Experience Questionnaire: development and validation of a measure for assessing recuperation and unwinding from work. Journal of occupational health psychology, 12(3), 204. doi: 10.1037/1076-8998.12.3.204
